# Autologous adipose-derived regenerative cell therapy modulates development of hypertrophic scarring in a red Duroc porcine model

**DOI:** 10.1186/s13287-017-0704-1

**Published:** 2017-11-15

**Authors:** Philippe Foubert, Diana Zafra, Mike Liu, Rohit Rajoria, Damian Gutierrez, Mayer Tenenhaus, John K. Fraser

**Affiliations:** 1 Cytori Therapeutics Inc, 3020 Callan Road, 92121 San Diego, CA USA; 2 Absorption Systems, San Diego, CA USA; 3 UCSD Medical Center, University of California, San Diego, CA USA

**Keywords:** Adipose-derived regenerative cells, Hypertrophic scarring, Wound healing, Inflammation

## Abstract

**Background:**

Effective prevention and treatment of hypertrophic scars (HTSs), a common consequence of deep-partial thickness injury, remain a significant clinical challenge. Previous studies from our group have shown that autologous adipose-derived regenerative cells (ADRCs) represent a promising approach to improve wound healing and, thereby, impact HTS development. The purpose of this study was to assess the influence of local delivery of ADRCs immediately following deep-partial thickness cutaneous injury on HTS development in the red Duroc (RD) porcine model.

**Methods:**

Bilateral pairs of deep-partial thickness excisional wounds (2 mm depth; 58 cm^2^ area) were created using an electric dermatome on RD pigs (n = 12). Autologous ADRCs were isolated from the inguinal fat pad and then sprayed directly onto the wound at a dose of 0.25 × 10^6^ viable cells/cm^2^. The paired contralateral wound received vehicle control. Wound healing and development of HTS were assessed over 6 months using digital imaging, quantitative measurement of skin hardness and pigmentation, and histology.

**Results:**

Data showed that ADRC treatment led to reduced scar hyperpigmentation compared to control (*p* < 0.05). Using the Durometer, at 2 and 6 months post-injury, skin hardness was 10–20% lower in ADRCs-treated wounds compared to control vehicle (*p* < 0.05). A similar trend was observed with the skin fibrometer.

ADRC treatment promoted more normal collagen organization, improvement in the number of rete ridges (*p* < 0.01), longer elastic fiber length (*p* < 0.01), and reduced hypervascularity (blood vessel density; *p* < 0.05). ADRC treatment was associated with modulation of IL-6 expression within the wound/scar with upregulation 2 weeks after injury (wound healing phase) and downregulation at 2 months (early scarring phase) post-treatment compared to control

**Conclusions:**

These findings support the potential therapeutic value of autologous ADRC administration for reduction of HTS development following deep-partial cutaneous injury.

**Electronic supplementary material:**

The online version of this article (doi:10.1186/s13287-017-0704-1) contains supplementary material, which is available to authorized users.

## Background

The wound-healing process is complex, requiring tight coordination of several cell types and numerous factors to ensure orderly, successful progression through the stages of healing leading to physiological repair [1]. While the development of hypertrophic scarring (HTS) is incompletely understood, it appears that certain injuries, particularly deep cutaneous wounds may overwhelm endogenous repair mechanisms leading to development of pathological scarring [2, 3]. HTS resulting in significant aesthetic disfigurement and functional impairment is relatively common following deep-partial cutaneous injuries [3]. HTS is associated with chronic wound inflammation, increased collagen synthesis, and increased cell turnover leading to erythema, pain, contraction, pruritus, reduced range of motion, cosmetic sequelae, and impaired quality of life [3]. Several methods have been proposed to reduce formation of HTS with mixed success [2, 4].

Regenerative medicine holds considerable promise to repair damaged tissues and organs and restore functionality. In this regard, the stromal vascular fraction (SVF) of adipose tissue has emerged as an innovative cell-based strategy to promote tissue repair [5–7]. The regenerative capacity of SVF is likely derived from the heterogeneity of its cellular constituents providing several avenues to improve tissue repair [6]. In this regard, a number of preclinical and clinical studies have reported that SVF, also referred to as adipose-derived rcgenerative Cells or ADRCs, modulates inflammation [8–11], angiogenesis [9, 11–13], and tissue fibrosis [14–19]. In our previously reported full-thickness burn studies in a Göttingen porcine model, we were able to show that autologous ADRC delivery improved wound angiogenesis and epithelialization [20–22]. While encouraging, our prior work was limited to assessment of short-term wound healing (up to 4 weeks post-injury) and did not address long term sequelae, such as HTS. Based on this evidence, we initiated a study to examine the effects of ADRCs on development of HTS in red Duroc (RD) pigs, a strain recognized to develop HTS in a fashion similar to humans [23, 24].

## Methods

### Animals

This study was conducted in compliance with the Animal Welfare Act, the implementing Animal Welfare Regulations and in accordance with the principles of the Guide for the Care and Use of Laboratory Animals. The study was conducted according to a research protocol reviewed and approved by Absorption Systems Institutional Animal Care and Use Committee (IACUC) for the designated Association for Assessment and Accreditation of Laboratory Animal Care International (AAALAC) accredited research facility. Twelve female pure RD pigs (40–60 kg) were purchased from Fresno State Swine Unit (Fresno, CA, USA). Animals were identified by ear tags, cage cards, and/or color identifiers in the animal’s back. The study animals were observed at least twice daily for signs of illness or distress, and any such observations were promptly reported to the veterinarian staff.

### Anesthesia and analgesia

Animals were fasted overnight prior to the use of anesthesia. Animals were anesthetized via an intramuscular injection of a cocktail containing ketamine (20 mg/kg), xylazine (2 mg/kg), and atropine (0.04 mg/kg). Upon loss of responsiveness and spontaneous movement, the animal was intubated and maintained on isoflurane (1 to 5%) in oxygen (1 to 3 L/min). Animals were ventilated as necessary. Heart rate, respiratory rate, oxygen saturation (SPO_2_), inspired/end tidal CO_2_, body temperature, and anesthetic depth were continuously monitored.

### Porcine model of deep-partial thickness excisional wounds

Following antiseptic preparation, two pairs of bilateral excisional cutaneous wounds (approximately 2 mm depth; 7.6 cm × 7.6 cm) were created in the flanks of each animal using a Zimmer dermatome set at a depth of 0.02 inch (0.5 mm) for four consecutive passes. Excisional biopsies (approximately 2 × 2 cm) were collected from each harvested skin layer in order to verify the thickness of each excision. In order to be acceptable for inclusion in the study each wound had to have a cumulative depth of 1.8–2.7 mm with no evidence of injury extending into subcutaneous adipose tissue (characteristic of full-thickness injury). In order to ensure that each pair of wounds within the same animal was matched at baseline, any wound pair for which the difference in cumulative wound depth was greater than 0.4 mm was excluded from the study. Only one (1) wound pair out of 12 did not meet these criteria. A multilayer dressing was applied to protect the wounds from infection and mechanical damage; Layer 1 (contact with the wound) Mepilex® Foam Dressing (Mölnlycke Health Care, Gothenburg, Sweden); layer 2 Ioban2™ (3 M Corporation, Maplewood, MN, USA), layer 3 stockinet (cotton wrap); layer 4 protective jacket (Sullivan Supplies, Houston, TX, USA). Bandaging was changed once every 5–7 days up to approximately week 4–5 when wounds were fully epithelialized. Three animals were euthanized at 2 weeks post-injury and a further three at 2 months post-injury for assessment of histology and expression of inflammatory growth factors. The remaining six animals were maintained up to 6 months post-injury.

### Adipose-derived regenerative cell (ADRC) isolation

Following wound injury (within 2 hours), adipose tissue (30–50 g) was excised from the inguinal fat region of the animals (while under general anesthesia) and isolated as previously described using Celase® enzyme [20–22]. ADRC yield and viability was determined using the NucleoCounter® NC‐100™ (ChemoMetec, Lillerød, Denmark), as previously described [22, 25].

### Flow cytometric characterization of ADRCs

Freshly isolated ADRCs were resuspended in staining buffer [0.2% bovine serum albumen (BSA) in phosphate-buffered saline (PBS); BD Biosciences, San Diego, CA, USA] and then stained with the following antibodies CD45-FITC (clone K252.1E4; Bio-Rad, Hercules, CA, USA), CD31-PE (clone LCI-4; Bio-Rad), CD90-PerCP-Cy™5.5 (clone 5E10; BD Biosciences, San Jose, CA, USA) and CD146-PE (P1H12; BD Biosciences). After 20 minutes incubation at 4 °C, cells were washed twice in staining buffer and fixed using a BDFACS Lysis Solution (BD Biosciences). Acquisition and analysis of the cells were performed on FACSAria using FACSDiva software.

### Clonogenic assay

Freshly isolated ADRCs were plated in six-well plate at low density (100 cells/cm^2^) in DMEM/F12 containing 10% fetal bovine serum (FBS). After 12–14 days, cells were rinsed with PBS, fixed with formalin, and stained with hematoxylin solution (Hemacolor kit; EMD Millipore, Billerica, MA, USA). Colonies containing > 50 fibroblast colony-forming units (CFU-F) were counted. CFU-F frequency was calculated by dividing the number of colonies by the number of seeded cells.

### Delivery of ADRCs onto the wound site

Freshly isolated ADRCs (total volume of 0.5 mL) were resuspended in lactated Ringer’s (LR) solution and delivered (within 2 hours of injury) onto the wound surface (approximately 58 cm^2^) at a dose of 0.25 × 10^6^ per cm^2^ using a spray system (nasal spray device, LMA-North America, San Diego, CA, USA), as previously reported [21]. Animals were slightly rotated on their side so that the wound surface was flat to ensure even distribution of ADRCs or control. Finally, cells were allowed to adhere onto the wound site for 3–5 minutes prior to wound covering. Each animal served as its own control; for each animal, wounds on the left flank were treated with ADRCs whereas those on the right flank received control LR solution.

### Planimetry wound imaging

Digital imaging of wounds was conducted on day 0 (post-injury), day 7, 14, 21, 28, 35, 60, 90, 120, and 180 post-injury. All wounds were subjected to high-quality digital imaging using the SilhouetteStar™ Wound Camera (ARANZ Medical, Christchurch, New Zealand). Wound images were then reviewed and total wound area was measured using the SilhouetteStar™ System.

### Wound histology

At day 60 (2 months) and 180 (6 months) post-injury, three (3) full-thickness specimens (approximately 3 cm × 1 cm) were harvested from across the scar. Samples were then fixed in 10% neutral-buffered formalin (NBF), embedded in paraffin, sectioned (5 μm), and stained with hematoxylin and eosin (H&E), Masson Trichrome and Verhoeff–van Gieson (VVG) stain. Entire slides were then digitally scanned using the Aperio ScanScope AT2 slide scanner (Aperio Technologies, Vista, CA, USA). Slides were viewed and analyzed using the ImageScope viewer (Aperio Technologies).

### α-SMA immunohistochemistry

Tissue specimens were fixed in 10% normal buffered formalin and subsequently embedded in paraffin. Tissue sections were subjected to an antigen retrieval step then incubated with primary polyclonal rabbit alpha-smooth muscle actin (α-SMA) (5 μg/ml, Abcam, Cambridge, MA, USA) antibody. alkaline phosphatase (AP)-based detection of the primary antibody was performed using a Vectastain ABC-AP kit (Vector Laboratories, Burlingame, CA, USA) according to the manufacturer’s instructions, followed by nuclear staining with Harris hematoxylin. As controls, tissue sections were stained as described above without adding primary antibody. α-SMA staining was quantified using ImageScope analysis software (Microvessel Analysis Algorithm; Aperio Technologies). Vascular smooth muscle cells were distinguished from other α-SMA-positive cells (such as myofibroblasts) on the basis of morphology.

### Skin hardness using the skin fibrometer and durometer

The SkinFibrometer (Delfin Technologies, Kuopio, Finland) was briefly pressed against the skin and the contact pressure was registered. The skin and the underlying upper subcutis resist the deformation and the induration value in newtons (N) was determined. The probe was briefly pressed against the skin for five consecutive times at four different sites within the scar area. In addition, a digital Rex Gauge Durometer (model DD-4, type 0, Rex Gauge Company Inc., Glenview, IL, USA), without a foot attachment was used to monitor scar hardness. During measurements, the durometer was rested by gravity against the skin at four different sites within the scar area. Tissue induration measurements were performed and recorded at approximately 2 and 6 months post-injury.

### Protein isolation and expression levels

Wound specimens were lysed using total protein extraction buffer (Thermo Fischer Scientific). Total protein content was determined using the BCA assay kit (Thermo Fischer Scientific). Interleukin-6 (IL-6) and tumor necrosis factor alpha (TNF-α) protein levels were determined in lysates of whole wounds (100 μg) using Quantikine porcine IL-6 and TNF-α kits (R&D Systems, Minneapolis, MN, USA). Assays were performed in accordance with the manufacturer’s instructions.

### Statistical analysis

Results are expressed as means ± standard error pf the mean (SEM). Comparisons between two groups were performed using a paired *t* test (two-tailed) (GraphPad Prism version 6.05; GraphPad Software, San Diego, CA, USA). A value of *p* < 0.05 was considered significant.

## Results

### Adipose-derived regenerative cells isolation, viability and characterization

ADRCs were successfully isolated from the inguinal fat pads. An average of 1.65 × 10^6^ ± 0.4 × 10^6^ ADRCs was obtained per gram of processed adipose tissue (range: 1.2 × 10^6^ –2.2 × 10^6^ cells/g tissue). The mean cell viability was 97.2% ± 0.4%; ranging from 96.7% to 97.6%. Flow cytometric evaluation of these cells showed that leukocytes (CD45^+^) comprised an average of 9.8% of ADRCs. Endothelial cells (CD31^+^/CD45^-^) averaged 5.7% of ADRCs and stromal cells (fibroblast and fibroblast–like; CD90^+^/CD45^-^) averaged around 68.9%. Smooth muscle-related cells (mural cells; CD31^-^/CD146^+^/CD45^-^) accounted for around 26.1% of ADRCs. In addition, using the CFU-F assay, the frequency of stromal progenitor/stem cells averaged 4.8% (Additional file 1: Figure S1).

### Wound-healing characteristics and HTS development following deep-partial thickness injury

**Fig. 1:**
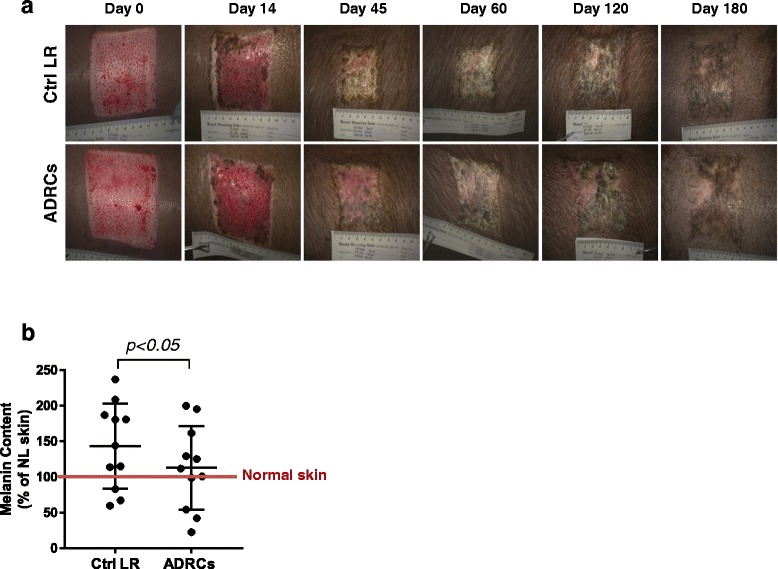
Wound-healing characteristics and HTS development in ADRCs- and control-LR treated wounds. This study consisted of six female red Duroc (RD) pigs each with two pairs of excisional wounds (2 mm depth). Each animal served as its own control. **a** Representative digital images of LR and ADRC-treated wound showing healing progression and HTS development up to 6 months post-injury. **b** Quantification of melanin content with the mexameter. 10–15 measurements were recorded within the scar area and surrounding normal skin. Normalization of skin pigmentation in control LR and ADRCs-treated wounds at 6 months post-injury was calculated. Results are presented as mean ± SEM. n = 6 animals; n = 11 wound pairs. ^*^ *p* < 0.05 versus LR control vehicle. *ADRCs* adipose-derived regenerative cells *Ctrl* control, *LR* lactated Ringer’s solution

Digital imaging showed that all wounds were fully epithelialized by day 45 (Fig. 1a). Although challenging using digital imaging, no significant difference in wound epithelialization was observed between control LR- and ADRC-treated wounds. Similarly, planimetry using the SilhouetteConnect™ software to determine wound contraction showed no significant difference between ADRCs and control LR-treated wounds (data not shown). However, it should be noted that evaluation of wound contraction was complicated by the fact that as the wound was contracting the animal as a whole was growing, leading to a net increase in scar size over the 6-month duration of the study.

### ADRC delivery improves scar pigmentation

Alterations in pigmentation, both hyper- and hypopigmentation, are frequently present in HTS [26]. The effect of ADRC treatment on pigmentation at 6 months post-injury was assessed in each wound using 15 locations across the entire site. The majority of wound pairs were predominantly hyperpigmented with small areas of hypopigmentation (Fig. 1a) while a small number were predominantly hypopigmented. This correlated directly with wound depth with deeper wounds tending to exhibit hypopigmentation rather than hyperpigmentation. Despite this variation, pigmentation in the ADRC-treated wounds was closer to that of uninjured adjacent skin than that of the control member of the wound pair (Fig. 1b; *p* < 0.05).

### ADRC delivery tends to reduce scar tissue hardness

**Fig. 2:**
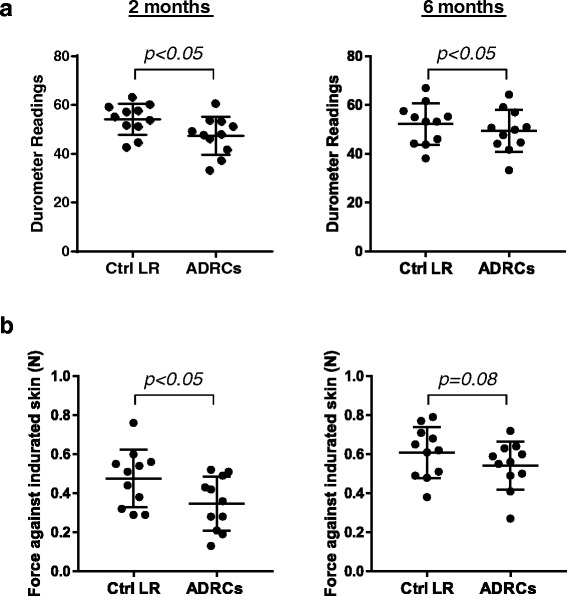
Effect of ADRCs on tissue hardness. Tissue hardness using the durometer (**a**) and fibrometer (**b**) was recorded at 2 and 6 months post-injury. Each line links the data points for a paired set of wounds. *n* = 6 animals; *n* = 11 wound pairs. ^*^ *p* < 0.05 versus LR control vehicle. *ADRCs* adipose-derived regenerative cells *Ctrl* control, *LR* lactated Ringer’s solution

Skin hardness of each wound was measured at 2 and 6 months post-injury/treatment using two non-invasive devices, the durometer and the fibrometer. Data obtained with the durometer showed reduced skin hardness (approximately 10–20% lower) in ADRCs-treated wounds (2 months: 33 ± 2.3 vs. 42.5 ± 1.9; 6 months: 33.2 ± 2.6 vs. 38.3 ± 2.6; *p* < 0.05 for both time points; Fig. 2a). A similar pattern was observed with the fibrometer (Fig. 2b) although the data with this device approached, but did not reach, statistical significance at 6 months (2 months: 0.34 ± 0.04 vs. 0.47 ± 0.04 N, *p* < 0.05; 6 months: 0.54 ± 0.03 vs. 0.6 ± 0.04 N, *p* = 0.08).

### ADRC delivery improves epidermal remodeling

**Fig. 3:**
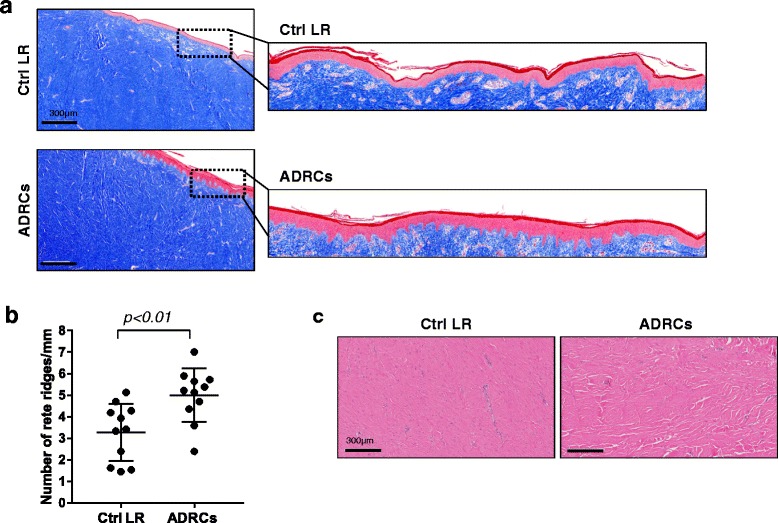
Effect of ADRCs on collagen organization and epithelial maturation. **a** Masson Trichrome staining performed on biopsies collected at 6 months post-injury. Inset: magnification showing epithelial flattening and maturation (presence of rete ridges) in LR and ADRCs-treated wound, respectively. **b** Quantification of rete ridges in LR- and ADRCs-treated wounds. **c** H&E staining performed at 6 months post-injury showing collagen organization in LR and ADRCs-treated wounds. Results are presented as mean ± SEM. n = 6 animals; n = 11 wound pairs. ^**^ *p* < 0.01 versus LR control vehicle. *ADRCs* adipose-derived regenerative cells *Ctrl* control, *LR* lactated Ringer’s solution

Flattening of the epidermis through loss of rete ridges, which play a key role in anchoring the epidermis to the dermis of healthy skin, is also characteristic of HTS [24, 27]. Quantitative analysis of rete ridge frequency in treated and control wound pairs demonstrated that the number of rete ridges was, on average, 48% greater in ADRCs-treated wounds compared to control LR (5.2 ± 1.4 vs. 3.5 ± 1.4 rete ridges per mm, respectively; *p* < 0.01) (Fig. 3a and b).

### ADRC delivery improves collagen deposition

Aberrant collagen orientation and bundle morphology are characteristic features of HTS [3, 27]. The most common criteria for the analysis collagen structure are the orientation and the thickness of the collagen bundles. In human HTS, collagen is discretely fibrillar with densely packed thin fibers running parallel to the epidermis. To investigate the effect of ADRCs on collagen organization, histochemical analysis of tissue sections was performed on biopsies collected 6 months post-injury. In LR-treated wounds, collagen fibers were densely packed and followed a parallel orientation to the epidermis (Fig. 3c). By comparison, ADRC-treated wounds exhibited thicker collagen bundles with larger bundle spacing (Fig. 3c).

### ADRC delivery at time of injury reduces scar tissue vascularization

**Fig. 4:**
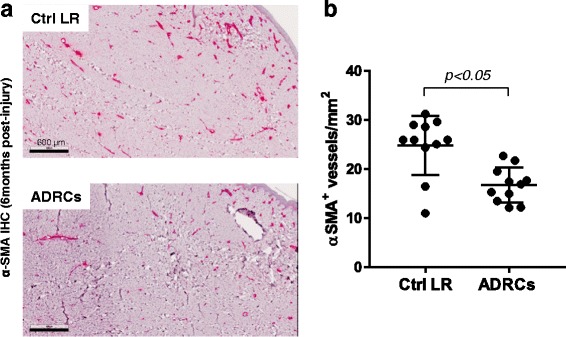
Effect of ADRCs on scar vascularization. **a** Biopsies collected at 6 months post-injury from LR and ADRCs-treated wounds were stained for α-SMA. Representative photomicrographs of sections stained with α-SMA. **b** Microvessel density (MVD) was quantified using automated analysis of digitally scanned slides. Results are presented as mean ± SEM. n = 6 animals; n = 11 wound pairs. ^*^ *p* < 0.05 versus LR control vehicle. *ADRCs* adipose-derived regenerative cells *Ctrl* control, *LR* lactated Ringer’s solution; α-SMA, alpha-smooth muscle actin

It is well-recognized that HTS is associated with hypervascularity [28]. To investigate the effect of ADRCs on microvessel density within the scar tissue, immunohistochemical analysis of tissue sections was performed on biopsies collected at 6 months post-injury using α-SMA, a recognized marker for smooth muscle in blood vessel walls. Digital quantification showed that microvascular density (MVD) was 27% lower in ADRC-treated wounds compared to LR-treated wounds (17.6 ± 4.7 vs. 24.4 ± 6 vessels per mm^2^, respectively; *p <* 0.05) (Fig. 4).

### ADRC delivery enhances elastic fibers length

Elastic fibers are important components of skin that are frequently lacking in HTS [29].

**Fig. 5:**
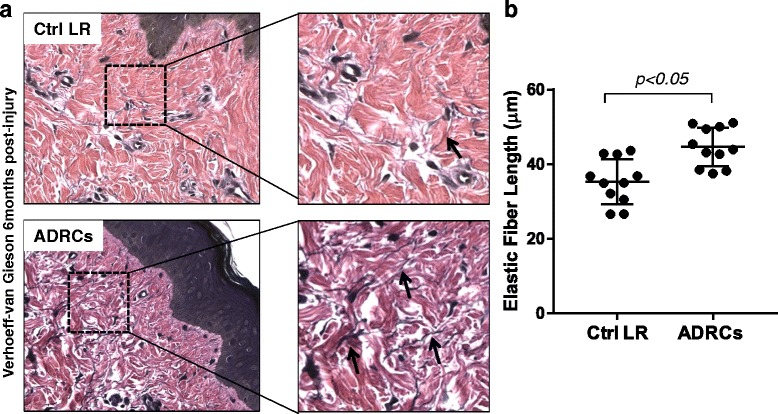
Effect of ADRCs on elastic fiber length. **a** Biopsies collected at 6 months post-injury from LR and ADRCs-treated wounds were stained for Verhoeff-van Gieson (VVG). Representative photomicrographs of sections stained for VVG. Inset: magnification elastic fibers (*black arrows*) **b** Elastic fiber length was quantified using the measuring tool of the Aperio ImageScope Software. Results are presented as mean ± standard error of the mean (SEM). n = 6 animals; n = 11 wound pairs. ^*^ *p* < 0.05 versus LR control vehicle. *ADRCs* adipose-derived regenerative cells *Ctrl* control, *LR* lactated Ringer’s solution

To investigate the effects of ADRCs on elastic fibers regeneration, specimens collected at 6 months were stained with the VVG solution. The mean elastic fiber length was significantly greater (approximately 23%) in ADRC-treated wounds compared to control LR-treated wounds (43.9 ± 5.4 vs. 35.5 ± 5.8 μm, respectively; *p* < 0.05) (Fig. 5).

### ADRC delivery modulates inflammation

**Fig. 6:**
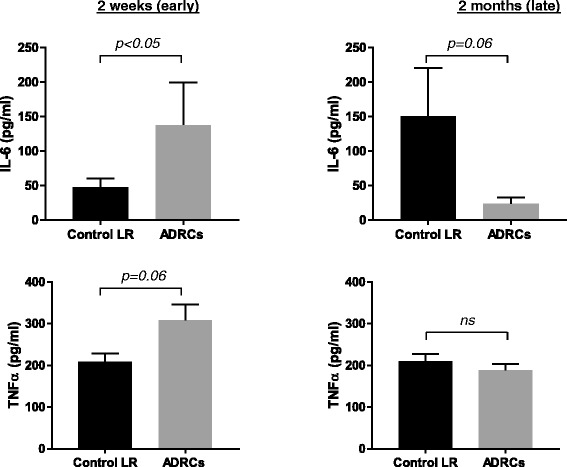
Effect of ADRCs on wound and early scar inflammation. Quantification of IL-6 and TNF-α protein levels in LR- and ADRC-treated wounds at 2 weeks (*n* = 3 animals) and 2 months (*n* = 6 animals) post-injury. Proteins were extracted and wound lysates were assayed for IL-6 and TNF-α using a porcine-specific enzyme-linked immune sorbent assay (ELISA) kit. Results are presented as mean ± SEM. n = 3-6 animals; n = 6-11 wound pairs. ^*^ *p* < 0.05 versus LR control vehicle. *ns* non-significant. *ADRCs* adipose-derived regenerative cells *Ctrl* control, *IL-6* interleukin-6, *LR* lactated Ringer’s solution. *TNF-α* tumor necrosis factor alpha

Deregulation/disruption of the inflammatory process is also associated with HTS formation [4, 30]. To further investigate the effects of ADRCs on the inflammatory process during wound healing and HTS formation. IL-6, TNF-α and IL-10 cytokines levels were quantified during the wound-healing phase (at 2 weeks after injury) and during early HTS development (2 months post-injury) post-injury (n = 3 animals for each time point). As shown in Fig. 6, at 2 weeks post-treatment, ADRCs-treated wounds exhibited higher expression of both IL-6 (2.7-fold; *p* < 0.05) and TNF-α (1.5-fold; *p* = 0.06) compared to control. By contrast, at 2 months post-treatment, IL-6 cytokine levels were lower (4.5-fold; *p* = 0.06) in ADRC-treated wounds compared to control. No significant difference in TNF-α levels was observed at 2 months post-treatment between ADRCs- treated and control wounds (Fig. 6). IL-10 was not detected at either time point in either treated or control wounds (data not shown).

## Discussion

HTS is a common complication of thermal burn injury frequently resulting in significant functional impairment and/or cosmetic defects [3]. The apparent ability of adipose-derived cells [such as SVF/ADRCs and adipose-derived stromal cells (ADSCs)] to decrease wound inflammation and favor tissue repair/regeneration at the wound site makes them an attractive therapeutic option for amelioration of scar formation. Several studies have reported that female and castrated male RD pigs exhibit many of the features of HTS observed in humans following cutaneous injury [23, 24, 31]. For this reason, they are increasingly used as a model of human HTS. While the RD pig is recognized as one of the best available model of HTS, it is not completely representative of human HTS [23, 32]. For example, human hypertrophic scars are invariably raised; this characteristic is not prominent in the RD swine. Nevertheless, in the current study, the model exhibited several features of human HTS including skin hardening, abnormal pigmentation, flattening of the epidermis, hypervascularity, and dysregulation of inflammatory mediators. Each of these parameters was modulated by topical treatment with autologous ADRCs.

In particular, our data showed a significant increase in the frequency of rete ridges within ADRCs-treated wounds. Rete ridges, also referred to as rete pegs, play a key role in anchoring the epidermis to the dermis thereby helping to protect the epidermis from injury as a result of shear force. Loss of rete ridges with a flattening of the epidermal/dermal junction is characteristic of HTS [24, 27]. In addition to this structural improvement, treated wounds also exhibited more normal vascularity (as evidenced by the frequency of smooth muscle-coated small vessels within the dermis), more normally organized collagen bundles, and longer elastic fibers. The increased length of elastic fiber is consistent with data showing reduced skin hardness in the treated wounds. These findings are generally consistent with data from other groups to assess scarring. For instance, Yun et al. have shown that subcutaneous injections of cultured ADSCs reduced scar contraction and improved scar color and pliability in Yorkshire swine, which do not develop HTS [33]. Furthermore, intralesional injection of ADSC in a rabbit ear model reduced α-SMA and collagen type I gene expression at day 35 post-treatment [34]. Similarly, Domergue et al. evaluated the effect of SVF and cultured ADSCs on pre-existing HTS in a nude mice model. Both treatments were associated with decreased skin thickness and collagen deposition at 2 weeks post-treatment [35]. Our data extend these observations by using a large animal model that is more similar to human HTS and by assessment of long-term outcome (6 months post-injury/treatment).

The inflammatory system plays a key role in wound healing, hypertrophic scar formation, and hyperpigmentation. In the early phase of healing inflammatory cell recruitment is critical to debride the wound site and to initiate angiogenesis, epithelial and matrix remodeling through expression of several cytokines (including IL-6, IL-10, and TNF-α) [36, 37]. In the early (proliferative) phase of wound-healing IL-6 promotes cell proliferation, angiogenesis and extracellular matrix (ECM) synthesis. However, several studies have shown that prolonged expression of IL-6 is associated with chronic inflammation and development of HTS [36, 38]. It has been shown that IL-6-trans-signaling-STAT3 pathway plays an integral role in HTS pathogenesis, mediating ECM production and dermal fibroblast proliferation [39]. In addition, IL-6 has been reported to be highly expressed in fibroblasts present in HTS tissue compared to normal fibroblasts [40]. Our data showed that, compared with control, ADRC treatment was associated with upregulation of IL-6 expression during the proliferative phase (at 2 weeks post-treatment) but a downregulation during the remodeling phase (2 months post-treatment). This is consistent with a pattern of accelerated early healing associated with reduced HTS formation and consistent with the observation of relative normalization of pigmentation in ADRCs-treated wounds. For example, it has been suggested that post-inflammatory effects play a key role in hyperpigmentation of scars. Further, Kim et al. have reported that cultured ADSCs mediate reduced activation and migration of melanocytes through expression of IL-6 [41].

## Conclusions

In conclusion, our data demonstrate that delivery of ADRCs at the time of injury modulates several characteristic features of HTS. It is important to note that our study is limited to deep-partial thickness excisional wounds in young healthy pigs. Therefore, it remains to be seen how these findings translate to HTS following severe burn injury or in patients of extremes of age or compromise. Second, this study assessed only a single dose of the cell therapy product. It may be that other dose regimens will prove more or less effective. Additional investigation will be required to assess the fate of injected cells though this will need to take into consideration the heterogeneous nature of the ADRC population. Overall, these findings expand upon our previous work and suggest that the improvement of healing following ADRC treatment extends beyond simple improvements in epithelialization to longer-term improvement in scar and skin function.

## Additional file

Additional file 1: Figure S1. Red Duroc ADRCs characterization. (**A**) Red Duroc pigs ADRCs flow cytometery. The presence of leukocytes, endothelial cells (ECs), stromal cells and mural cells was analyzed using CD45, CD31, CD90, and CD146 cell surface markers. (**B**) The fibroblastoid colony-forming unit (CFU-F) assay was performed to define the number of progenitor cells in animals subjected to burn injury alone and combined injury. n = 3–6 animals. (PPTX 272 kb)

## Abbreviations

ADRCs: Adipose-derived regenerative cells
ADSCs: Adipose-derived stromal cells
CFU-F: fibroblastoid colony-forming unit
ECM: Extracellular matrix
ELISA: Enzyme-linked immune sorbent assay
H&E: Hematoxylin & eosin
HTS: Hypertrophic scarring
IL-6: Interleukin-6
LR: Lactated Ringer’s solution
MVD: Microvascular density
N: Newton
NBF: Normal buffered formalin
RD: Red Duroc
SEM: Standard error of the mean
SVF: Stromal vascular fraction
TNF-α: Tumor necrosis factor alpha
VVG: Verhoeff–van Gieson
α-SMA: Alpha-smooth muscle actin
