# Supplementary figures and images for: Autologous adipose-derived regenerative cell therapy modulates development of hypertrophic scarring in a red Duroc porcine model
Source: Stem Cell Res Ther. 2017 Nov 15;8:261. doi: 10.1186/s13287-017-0704-1 (PMC5688645; doi:10.1186/s13287-017-0704-1)

## Slide 1
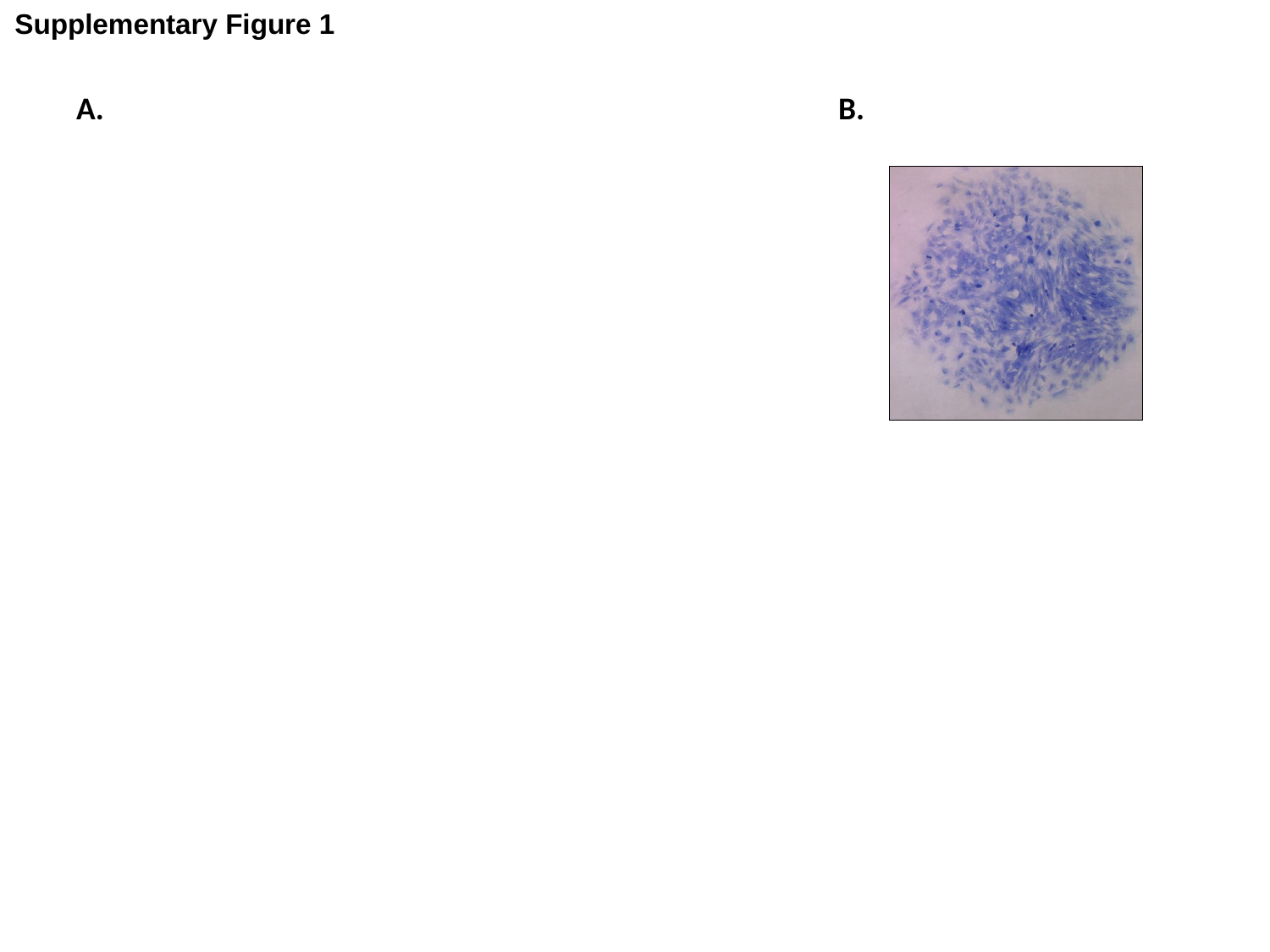

Supplementary Figure 1
A.
B.

Supplement: Additional file 1: Figure S1. — Red Duroc ADRCs characterization. (A) Red Duroc pigs ADRCs flow cytometery. The presence of leukocytes, endothelial cells (ECs), stromal cells and mural cells was analyzed using CD45, CD31, CD90, and CD146 cell surface markers. (B) The fibroblastoid colony-forming unit (CFU-F) assay was performed to define the number of progenitor cells in animals subjected to burn injury alone and combined injury. n = 3–6 animals. (PPTX 272 kb) [file 13287_2017_704_MOESM1_ESM.pptx]
